# Menstrual blood derived mesenchymal stem cells combined with Bushen Tiaochong recipe improved chemotherapy-induced premature ovarian failure in mice by inhibiting GADD45b expression in the cell cycle pathway

**DOI:** 10.1186/s12958-019-0499-2

**Published:** 2019-07-16

**Authors:** Fengyi Guo, Tian Xia, Yedan Zhang, Xiaotong Ma, Zhongrui Yan, Shaohua Hao, Yali Han, Ruihong Ma, Yuan Zhou, Xue Du

**Affiliations:** 1 Department of Obstetrics & Gynecology, General Hospital, Tianjin Medical University, NO.154, Anshan Road, Heping District, Tianjin, 300052 People’s Republic of China; 2 Reproductive Center, First Teaching Hospital of Tianjin University of Traditional Chinese Medicine, No. 88 Chang Ling Street, Xi Qing district, Tianjin, 300112 China; 3 State Key Laboratory of Experimental Hematology, Institute of Hematology and Blood Diseases Hospital, Chinese Academy of Medical Sciences and Peking Union Medical College, Tianjin, 300020 China

**Keywords:** Mesenchymal stem cells, Menstrual blood, Bushen Tiaochong recipe, Premature ovarian failure, Epirubicin, GADD45

## Abstract

**Background:**

To investigate the therapeutic effects of menstrual blood derived mesenchymal stem cells (MB-MSCs) combined with Bushen Tiaochong recipe (BSTCR) on epirubicin induced premature ovarian failure (POF) in mice.

**Methods:**

Twenty-four female C57BL/6 mice of 6–8 weeks were intraperitoneally injected with epirubicin to induce POF, and then they were randomized into 4 groups of 6 mice each and treated with PBS, MB-MSCs, BSTCR, and MB-MSCs combined with BSTCR, respectively. Six mice of the same age were used as controls. Vaginal smear, TUNEL and hematoxylin-eosin staining were to observe estrous cycles, ovarian cell apoptosis and follicles. Enzyme-linked immunosorbent analysis determined serum estradiol, follicle-stimulating hormone (FSH) and anti-Müllerian hormone (AMH) levels. RT-qPCR and Western Blot analysis were to determine GADD45b, CyclinB1, CDC2 and pCDC2 expressions.

**Results:**

Epirubicin treatment resulted in a decrease in the number of primordial, primary, secondary and antral follicles, an increase in the number of atretic follicles and ovarian cell apoptosis, a decrease in estradiol and AMH levels, an increase in FSH levels, and estrous cycle arrest. However, MB-MSCs combined with BSTCR rescued epirubicin induced POF through down-regulating GADD45b and pCDC2 expressions, and up-regulating CyclinB1 and CDC2 expressions. The combined treatment showed better therapeutic efficacy than BSTCR or MB-MSCs alone.

**Conclusions:**

MB-MSCs combined with BSTCR improved the ovarian function of epirubicin induced POF mice, which might be related to the inhibition of GADD45b expression and the promotion of CyclinB1 and CDC2 expressions. The combined treatment had better therapeutic efficacy than BSTCR or MB-MSCs alone.

## Background

Chemotherapy has the potential to increase the survival of patients with malignant tumors, but concerns have also been raised about chemotherapy-induced decrease in ovarian function in young patients. Cancer patients such as breast cancer, leukemia and lymphoma are usually diagnosed at young ages and the lifetime is prolonged after chemotherapy. However, amenorrhea and infertility caused by chemotherapy has gradually been paid attention to [1–3]. Anthracycline antibiotics such as doxorubicin and epirubicin are the first-line chemotherapeutic agents for the treatment of acute leukemia, lymphoma and breast cancer. Epirubicin is a cell cycle phase non-specific anthracycline, with maximal cytotoxic effects in the S and G2 phases, the mechanism of which as an antitumour drug forms a complex with DNA by intercalation between the DNA strands, thus inhibiting replication and transcription [4]. It also suppresses the activity of topoisomerase II and upregulates the level of reactive oxygen species (ROS) to kill cancer cells [5]. It was reported that anthracycline- and taxane-based chemotherapy was associated with a 40–60% risk of treatment-induced POF in breast cancer patients under the age of 40 years [6], but the mechanism of ovarian toxicity remains unclear. Doxorubicin could induce GC apoptosis and follicle damage [7, 8]. However, the ovarian toxicity of epirubicin has rarely been reported.

Studies have reported that epirubicin induces POF in humans. The classic fluorouracil, epirubicin, and cyclophosphamide (FEC) regimen induces menopause in 60% of breast cancer patients [9]. The National Cancer Institute of Canada adjuvant trial comparing cyclophosphamide, methotrexate, and 5-fluorouracil regimen (CMF) with FEC regimen indicated that the incidence of amenorrhea was slightly higher in FEC arm (51%) in comparison with the CMF arm (42.6%) [10]. In addition, the chemotherapy of breast cancer associated with epirubicin resulted in an incidence of amenorrhea of 23.08–44.87% [11]. Thus, the point of this study was to explore the mechanisms of ovarian toxicity of epirubicin. Besides, chemotherapy induced premature ovarian failure (POF) can cause serious long-term complications such as vasomotor symptoms, osteoporosis and cardiovascular diseases [12].

The mechanism of chemotherapy induced ovarian damage may be related to the apoptosis of granulosa cells (GCs), injury to growing follicles, consumption of primordial follicles, and damage to ovarian microenvironment [13–17]. However, no effective treatment is currently available for chemotherapy induced POF. In recent years, it has been reported that mesenchymal stem cells (MSCs) have the potential to repair POF as they can be differentiated into GCs, reduce GC apoptosis, prevent follicular atresia and maintain healthy follicles, and improve the renewal of germline stem cells and ovarian microenvironment [18–21]. We have previously found that MSCs derived from menstrual blood (MB-MSCs) could repair epirubicin induced damages to human ovarian GCs [22]. We have also found that in an epirubicin induced POF mice model, MB-MSCs could increase Estradiol and antimullerian hormone (AMH) levels and the number of primordial, primary, secondary and antral follicles and decrease the number of atretic follicles by improving the apoptosis of ovarian cells. However, the estrous cycle at 28 d of MB-MSCs transplantation showed no significant recovery. Therefore, a new strategy is needed to cooperate with MB-MSCs to improve the damaged ovarian function through a variety of ways. In traditional Chinese medicine (TCM) theory, Shen governs reproduction. Damage of Shen is thought to be the main etiology of POF. Studies have reported that Bushen, which means tonifying Shen, can improve POF [23–28]. Bushen Tiaochong recipe (BSTCR) is a traditional Chinese medicine prescription for tonifying Shen which is made up of nine kinds of Chinese herbs. It is capable of improving ovarian microenvironment and the expressions of follicle stimulating hormone receptor (FSHR) and insulin-like growth factors − 1 (IGF-1) mRNA and enhancing the efficacy of gonadotropin to GCs and the reactivity of GCs to gonadotropin. BSTCR can recover the ovarian reserve capacity via the brain derived neurotrophic factor (BDNF) pathway and improve the proliferation of ovarian GCs and the secretion of steroid hormones [23, 24]. Thus, it is expected that BSTCR maycoordinate with MB-MSCs to improve the ovarian function in epirubicin induced POF mice.

In the present study, we studied the effect and the relevant mechanisms of MB-MSCs combined with BSTCR on the ovarian function after epirubicin chemotherapy in mice and compared the efficacy of the combined treatment with MB-MSCs or BSTCR treatment, respectively.

## Methods

### Cell preparation

All protocols were approved by the Ethics Committee of Tianjin Medical University, and informed written consent was obtained from all participants. MB was collected from three healthy females, and MB-MSCs were isolated, identified and cultured as described previously [29]. MB-MSCs cultured to the third generation were seeded at a density of 2 × 10^5^ cells/well in 6-well plates in DMEM/F12 solution containing 10% FBS. When they were grown to 60% confluence, 600 μL of green fluorescent protein (GFP)-luciferase adenovirus suspension (10^7^ PFU/ml) (kindly provided by the Viral Laboratory of Hematonosis Hospital of Chinese Academy of Medical Sciences) was added. Fluorescence microscopy (Zeiss, Germany) showed that MB-MSCs were successfully labeled by GFP, and then they were suspended in PBS at a density of 5 × 10^5^ cells/100 μL.

### BSTCR preparation

All constitutes of BSTCR (Sheng Shilong Pharmaceutical. Co., Ltd., China) were decocted twice, and the two decoctions were mixed and boiled to prepare the final decoction with a concentration of 3 g crude drugs/mL.

### Establishment and grouping of animal models

Female C57BL/6 mice at 8 weeks (18-20 g) were purchased from Beijing Huafukang Biotechnology Co., LTD, (Beijing, China), and the vaginal smear showed that they had at least two consecutive normal estrous cycles of 4–5 days. All mice were fed ad libitum with sterile food and water at a controlled temperature of 21–24 °C on a 12 h light:12 h dark cycle. They were intraperitoneally injected with 0.01 mg/g epirubicin for 7 consecutive days, and vaginal smear was performed regularly each day. The animal model was successfully established if no estrous cycle was observed for 2 cycles (approximately 8 days).

Twenty-four POF mice were randomized into 4 groups of 6 mice each, which were treated with tail intravenous injection and gavage of PBS (E group); tail intravenous injection of 200 μL PBS containing 1 × 10^6^ GFP-labeled MB-MSCs (E + M group); 5 μL/g BSTCR gavage for 3 consecutive days (E + B group); MB-MSCs transplantation and BSTCR gavage as described above (E + M + B group), respectively. Six mice of the same age were used as controls, which were treated with intraperitoneal and tail intravenous injection and gavage of PBS. All of the experiments were repeated three times.

After MB-MSCs transplantation for 24 h, the homing of MB-MSCs was observed using a real-time imaging system in three mice in each group, and changes in estrous cycles were observed daily for 28 d in the rest. After that, they were sacrificed by cervical dislocation, and one ovary was collected for TUNEL and HE staining to observe ovarian in-situ apoptosis and follicle development, and the other ovary was collected for reverse transcription-quantitative polymerase chain reaction (RT-qPCR) and Western Blot analysis of GADD45b, CyclinB1, CDC2 and pCDC2 expressions.

Finally, 150 μL of peripheral blood was collected from the caudal vein for ELISA analysis of serum estradiol (E2), follicle-stimulating hormone (FSH) and anti-Müllerian hormone (AMH) levels.

### Live imaging of transplanted MB-MSCs in mice

After MB-MSCs transplantation for 24 h, mice were intraperitoneally anesthetized with 10% chloral hydrate (3 μL/g) (Kermel, China) and then intraperitoneally injected with D-fluorescein (150 μg/g) (Promega, USA). Ten minutes later, green fluorescence was observed with a live imaging system (Xenogen, USA).

### Enzyme-linked immunosorbent (ELISA) assay

After placed at room temperature for 2 h, mice blood was centrifuged at 2000 g for 20 min, and the supernatant was collected. The E2, FSH and AMH levels were measured using an ELISA kit (Cloud-Clone Corp, USA) according to the manufacturer’s protocol, and the results were analyzed using a Synergy H4 Hybrid Reader (Bio Tek, USA).

### Histopathology

Ovaries were collected and fixed in 4% paraformaldehyde for 24 h. Then they were embedded in paraffin and sliced into pieces of 5 μm thick for hematoxylin and eosin staining (H&E staining).

Follicles of every stage were classified and counted according to the following criteria. A primordial follicle was defined as an oocyte surrounded by a single layer of flattened squamous pregranulosa cells. A primary follicle was defined as an oocyte surrounded by a single layer of cuboidal granulosa cells. Secondary follicles had two or more layers of cuboidal granulosa cells with no visible antrum. Follicles were defined as antral follicles if they had an antral space with a cumulous granulosa cell layer. Follicles were classified as atretic if the oocyte was degenerating (convoluted, condensed) or fragmented. [18, 25].

### Terminal deoxynucleotidyltransferase-mediated deoxy-UTP nick end labeling (TUNEL) assay

One ovary was fixed in 4% formaldehyde for 24 h and embedded in paraffin. Then, it was sliced into pieces of 5 μm thick and dewaxed for the detection of in situ apoptosis using a TUNEL kit (Yeasen, China). Five random fields per section and five sections per tissue from a mouse were examined and analyzed in each experiment.

### RNA extraction and reverse transcription-quantitative polymerase chain reaction (RT-qPCR)

Total RNA from ovarian tissue was obtained using TRIzol regent (Life Technologies) and reverse transcribed to cDNA using ImProm-IITM Reverse Transcription Kit (Promega, Madison, USA). Primers for GADD45b consisted of 5′-GAGGCGGCCAAACTGATGAAT-3′ (forward) and 5′-CGCAGCAGA ACG ACT GGAT-3′ (reverse). Primers for CyclinB1 consisted of 5′-AAGGTGCCTGTGTGTGAACC-3′ (forward) and 5′-GTCAGCCCCATCATCTGCG-3′ (reverse). Primers for CDC2 consisted of 5′-AGAAGGTACTTACGGTGTGGT-3′ (forward) and 5′-GAGAGATTTCCCGAATTGCAGT-3′ (reverse). Primers for GAPDH consisted of 5′-AGGTCGGTGTGAACGGATTTG-3′ (forward) and 5′-TGTAGACCATGTAGTTGAGGTCA-3′ (reverse). The mRNA expression levels were measured on a 7900 real-time PCR instrument (Applied Biosystems) with the following amplification profile: 3 min at 94 °C followed by 30 cycles at 94 °C for 30 s, 55 °C for 30 s and 72 °C for 1 min. The relative expression level of mRNA was calculated using the 2^−ΔΔCт^ method adjusted by GAPDH as an internal control.

### Western blot analysis

The total protein was extracted from ovarian tissue according to the manufacturer’s instructions, and the total protein concentration was determined using the bicinchoninic acid assay (BCA; Solarbio, China). In each group, 10 μL of samples were electrophoresed on a sodium dodecyl sulfate-10% polyacrylamide gel and then transferred to polyvinylidene fluoride (PVDF) membrane (Bio-Rad, USA). The membrane was blocked in 5% defatted milk at room temperature for 2 h, and then incubated with the primary antibody against GADD45b (Santa Cruz, USA, 1:1000), CyclinB1 (Cell Signaling Technology, USA, 1:1000), CDC2 (Cell Signaling Technology, 1:1000), pCDC2 (Cell Signaling Technology, 1:1000) and β-actin antibody (Cell Signaling Technology, 1:1000) diluted in 1 × TBST containing 5% defatted milk overnight at 4 °C, and then with HRP labeled secondary antibody (Beyotime, China, 1:1000) diluted in 1 × TBST containing 5% defatted milk at room temperature for 2 h. The bands were visualised using an enhanced chemiluminescence detection system (PerkinElmer, USA) and exposed by ImageQuant LAS 4010 Control Software (GE, USA). Fluorescence intensity was quantitated using Image J (National Center for Biotechnology Information, National Institutes of Health, Bethesda, MD). The GADD45b, CyclinB1, CDC2 and pCDC2 expressions were normalized to β-actin expression.

### Statistical analyses

All continuous data were expressed as mean ± standard deviation and analyzed using SPSS 20.0 (SPSS Inc., USA). Statistical differences between two groups were analyzed by two-tailed unpaired t-test; whereas those among three or more groups were analyzed by one-way analysis of variance (ANOVA) and Tukey multiple comparison test. *P* < 0.05 was considered statistically significant.

## Results

### MB-MSCs migrated to pelvic organs of POF mice

**Fig. 1:**
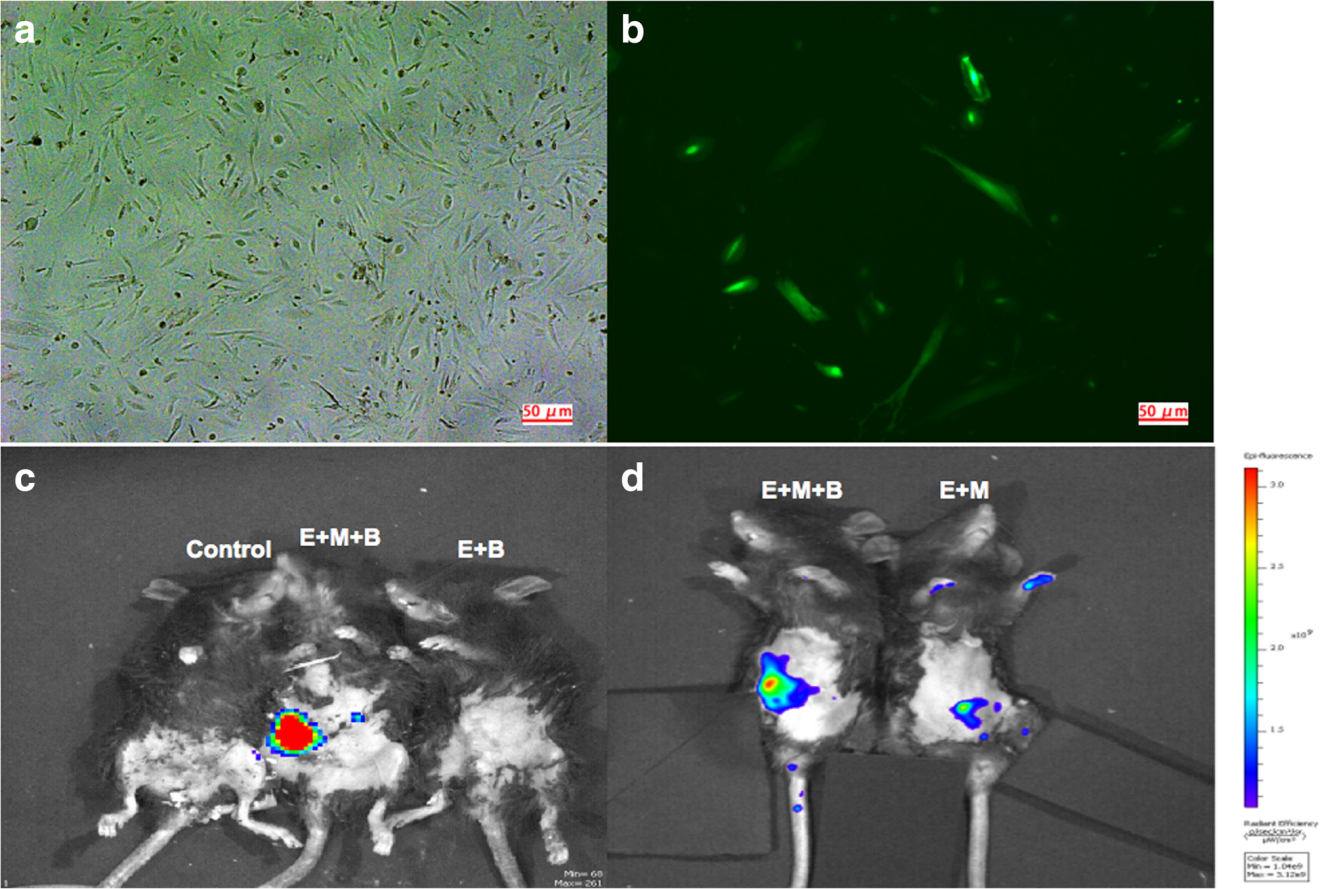
In vivo tracking of MB-MSCs. **a** Cultured MB-MSCs. **b** GFP-transfected MB-MSCs. ×40 magnification. Scale bars: 50 μm. **c-d** Twenty-four hours after tail vein transplantation, mice were screened by live imaging for in vivo tracking of GFP-positive cells

GFP labeled MB-MSCs were observed under a fluorescence microscope after transfection for 48 h (Fig. 1a and b) with a transfection rate of 20–30%. To elucidate the homing of MB-MSCs in vivo after tail intravenous transplantation, mice were sterilized and then screened by live imaging to track GFP (+) cells. GFP (+) cells were observed in pelvic organs 24 h after transplantation (Fig. 1c). Interestingly, stronger fluorescence was observed in E + M + B group than that in E + M group (Fig. 1d), which indicated that BSTCR might enhance the migration of MB-MSCs in mice. However, more evidence is needed to verify this result.

### The combined treatment increased body weight of POF mic

**Fig. 2:**
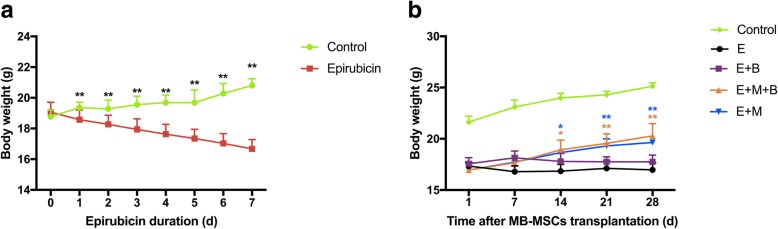
The effect of chemotherapy and different treatments on body weight. **a** Effect of epirubicin. **b** Effect of different treatments. *P < 0.05, **P < 0.01 v.s. E group

Mice showed hair loss, loss of appetite, and poor reactivity to external stimuli three days after injection with epirubicin. Body weight was monitored daily, and those mice injected with epirubicin had a significantly lower body weight than control mice (*P* < 0.01) (Fig. 2a).

MB-MSCs transplantation was given once upon the POF model was established successfully. Body weights in E + M group and E + M + B group were significantly increased from 14d onward after cell transplantation (*P* < 0.05, Fig. 2b). However, no significant difference was observed between E + B group and E group (*P* > 0.05, Fig. 2b). Besides, there was no significant difference between E + M group and E + M + B group (*P* > 0.05, Fig. 2b).

### The combined treatment improved estrous cycles of POF mice

**Fig. 3:**
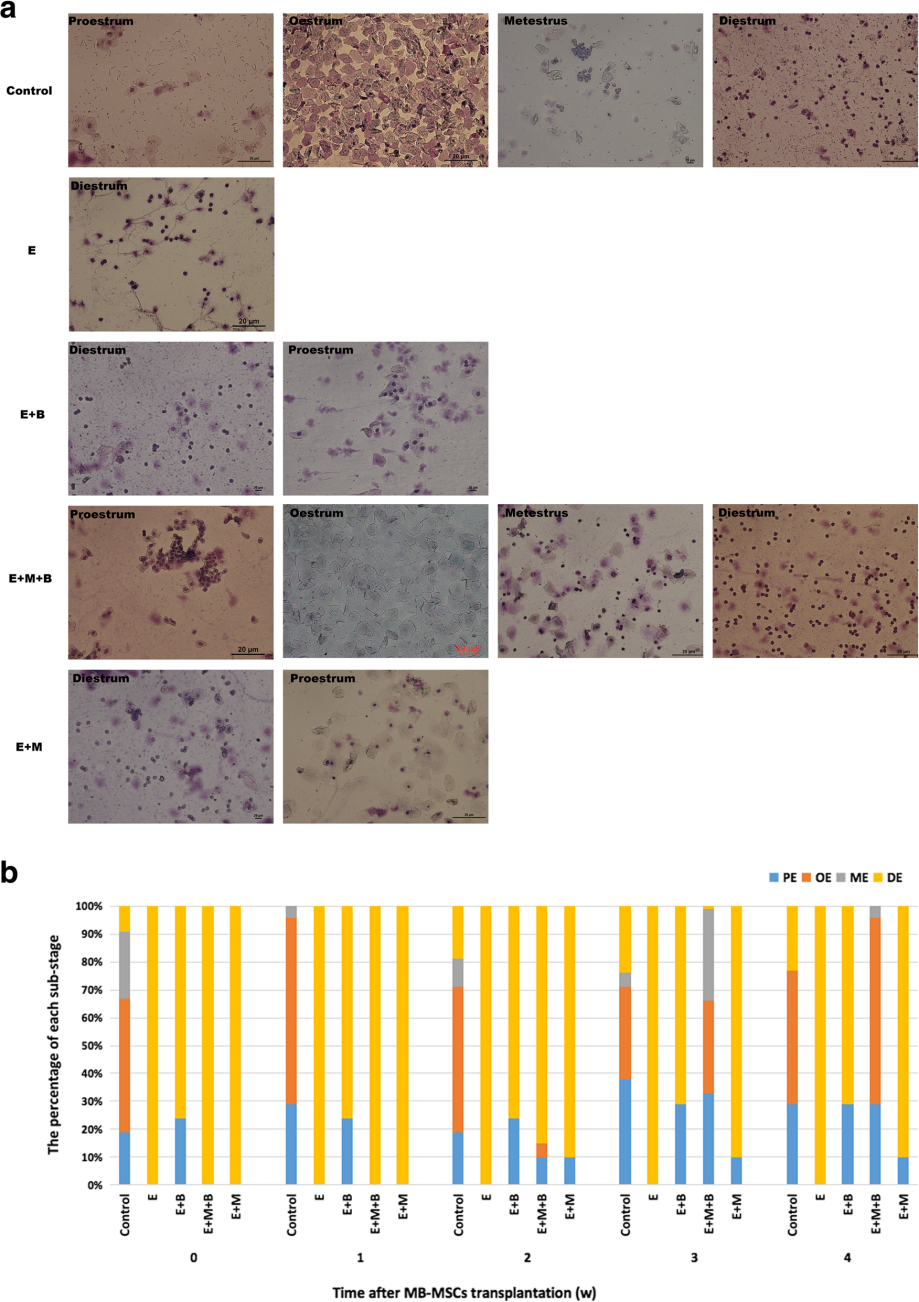
The effect of chemotherapy and different treatments on estrous cycles. **a** Vaginal smears in different groups. × 200 magnification. Scale bars: 20 μm. **b** The percentage of substages of proestrum (PE), oestrum (OE), metestrus (ME) and diestrum (DE) in different groups

The estrous cycle of mice can be divided into proestrum, oestrum, metestrus and diestrum. Compared with the control group, epirubicin treatment resulted in a longer estrous cycle, so that the estrous cycle was arrested in diestrum or proestrum after 7 d (Fig. 3a). Mice were observed for 2–3 consecutive estrous cycles (approximately 14 d), and it was found that those mice treated with epirubicin remained in diestrum or proestrum, indicating that epirubicin-induced POF mice model has been successfully established.

After 14 days of MB-MSCs transplantation, the estrous cycle was gradually recovered in E + M + B group and started cyclicity from 20 d onward. However, mice in other groups still showed no recovery on 28 d. The estrous cycles of mice in E group, E + M group and E + B group were still absent and remained in diestrum or proestrum (Fig. 3b).

### The combined treatment improved sex hormones secretion in POF mice

**Fig. 4:**
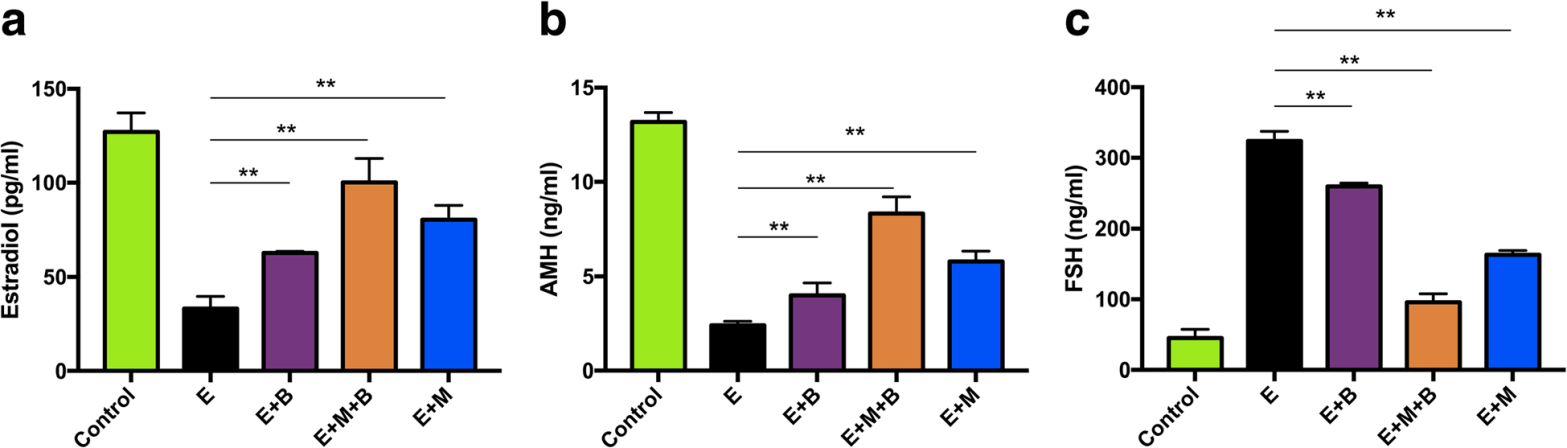
The effect of chemotherapy and different treatments on sex hormone secretion. **a** E2 levels. **b** AMH levels. **c** FSH levels. *P < 0.05, **P < 0.01 v.s. E group

Serum sex hormone levels were significantly different among groups. The levels of E2 and AMH of mice were significantly decreased after epirubicin injection (*P* < 0.01, Fig. 4a and b), while the level of FSH was significantly increased (*P* < 0.01, Fig. 4c). The levels of E2 and AMH in E + B group, E + M + B group and E + M group were significantly increased compared to E group (*P* < 0.01, Fig. 4a and b), while the level of FSH in E + B group, E + M + B group and E + M group was significantly lower than that in E group, respectively (*P* < 0.01, Fig. 4c). In addition, the levels of sex hormones in E + M + B group were almost significantly different from E + M group and from E + B group, respectively (E2: *P* > 0.05 v.s. E + M group; *P* < 0.01 v.s. E + B group. AMH: *P* < 0.05 v.s. E + M group; *P* < 0.01 v.s. E + B group. FSH: *P* < 0.01 v.s. E + M group; *P* < 0.01 v.s. E + B group).

### The combined treatment improved ovarian morphology in POF mice

In order to detect the effect of epirubicin administration on the total number of follicles in every stage, we randomly selected five slices of every ovary and classified and counted the follicles on the entire field of these five slices. Three ovaries from three mice were calculated in each group and the total follicles in each group were added and used for further analysis.

**Fig. 5:**
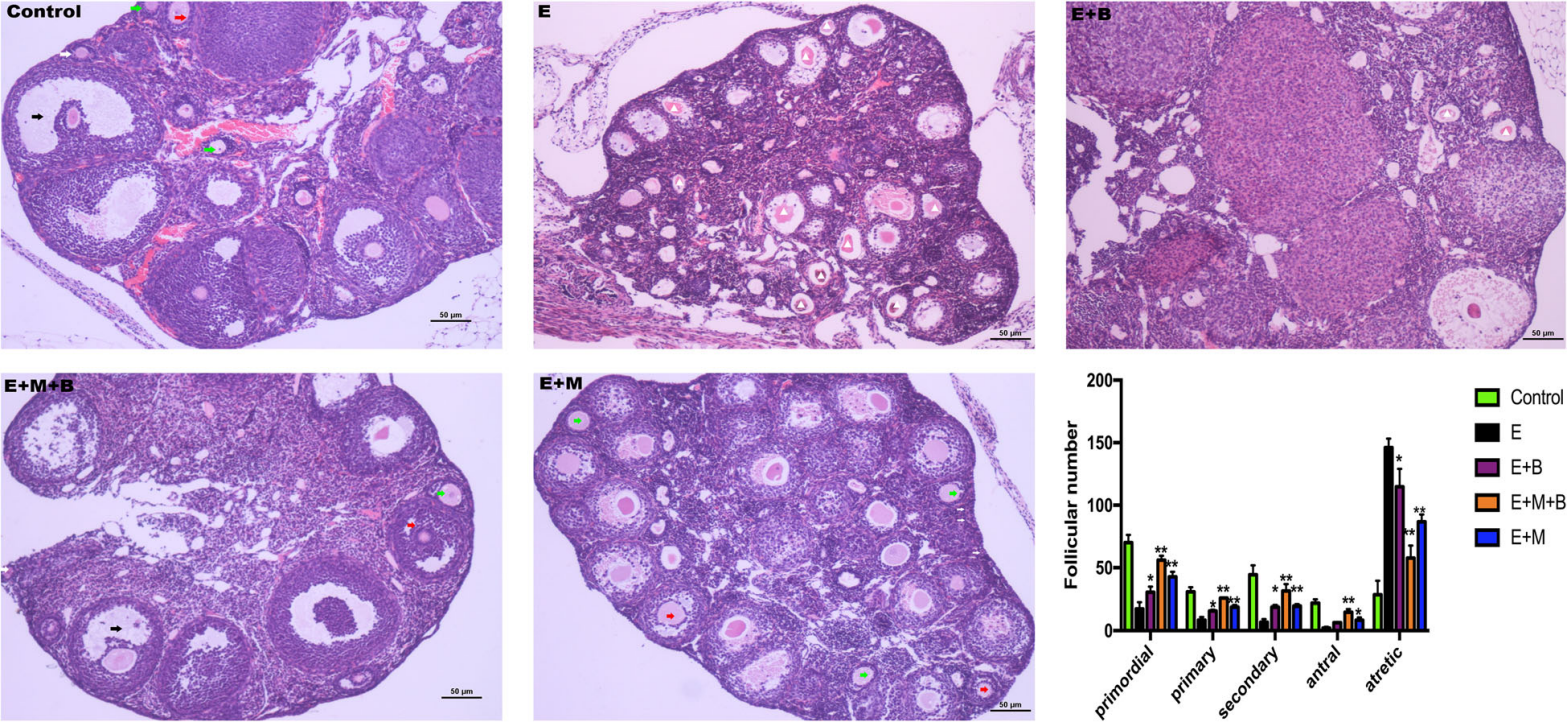
The effect of chemotherapy and different treatments on follicle development. a. Follicle number per ovary in different groups. White arrow: primordial follicles; green arrow: primary follicles; red arrow: secondary follicles; black arrow: antral follicles; white triangle: atretic follicles. *P < 0.05, **P < 0.01 v.s. E group. b HE staining of ovaries. × 100 magnification. Scale bars: 50 μm

Epirubicin exposure resulted in a decrease in the number of primordial follicles, primary follicles, secondary follicles and antral follicles (*P* < 0.01, Fig. 5), but an increase in the number of atretic follicles (*P* < 0.01, Fig. 5). However, the number of healthy follicles at all stages increased and the number of atretic follicles decreased in E + M + B group (*P* < 0.01, Fig. 5) and E + M group (*P* < 0.05, Fig. 5), respectively. A significant difference of primordial follicles, primary follicles, secondary follicles and atretic follicles was observed between E group and E + B group (*P* < 0.05, Fig. 5) except of antral follicles (*P* > 0.05, Fig. 5). Besides, E + M + B group indicated a more significant efficacy on follicular development than E + B group and E + M group, respectively (*P* < 0.05, Fig. 5).

### The combined treatment decreased ovarian cell apoptosis in POF mice

**Fig. 6:**
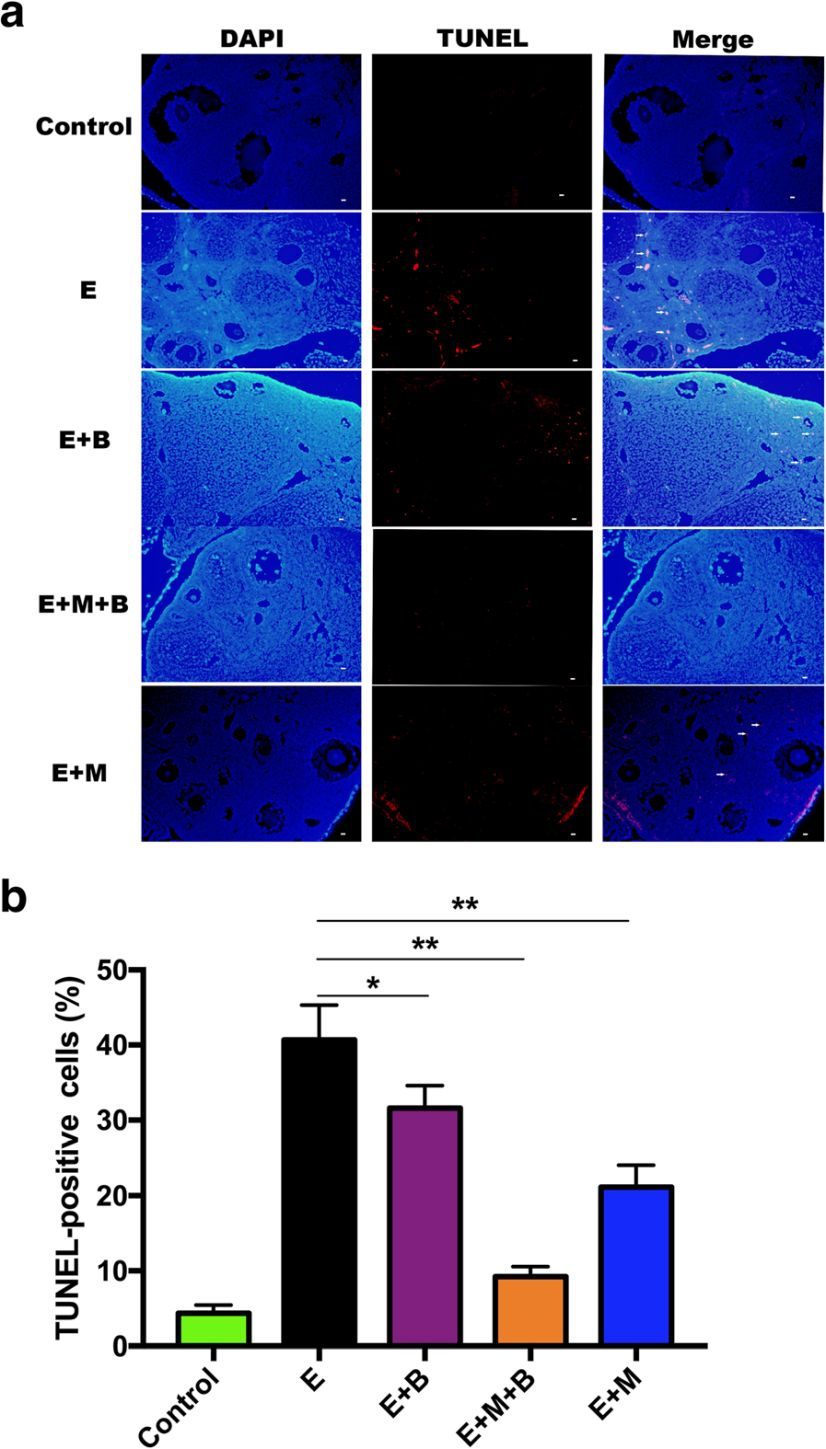
The effect of chemotherapy and different treatments on ovarian cell apoptosis. **a** Ovarian cell apoptosis was analyzed by TUNEL staining. Blue fluorescence indicated cell nucleus stained by 4′, 6-diamidino-2-phenylindole (DAPI). TUNEL-positive cells labeled red (Alexa Fluor 640) indicated cell apoptosis (white arrow). × 200 magnification. Scale bars: 50 μm. **b** Quantitative analysis showing the percentage of TUNEL-positive cells in each group (*n* = 3 per group). *P < 0.05 and **P < 0.01 v.s. E group

To investigate if epirubicin induces ovarian cell apoptosis and the effect of MB-MSCs and/or BSTCR treatment, ovarian tissue slides of each group were stained with TUNEL kit. The rate of TUNEL-positive cells was significantly higher in mice exposed with epirubicin compared to control group (*P* < 0.01, Fig. 6b). The rate of TUNEL-positive cells was significantly decreased in POF mice after treatment of MB-MSCs and/or BSTCR. In addition, E + M + B group showed a stronger inhibition of cellular apoptosis compared to E + B group (*P* < 0.01, Fig. 6b) and E + M group (*P* < 0.01, Fig. 6b), respectively. Besides, TUNEL-positive cells were only observed in granulosa cells and most of the apoptotic cells were observed in atretic follicles, which suggests that the increase of granulosa cell apoptosis may be one of the potential mechanisms of epirubicin-induced POF in mice.

### Ovarian expression of GADD45b, CyclinB1 and CDC2

**Fig. 7:**
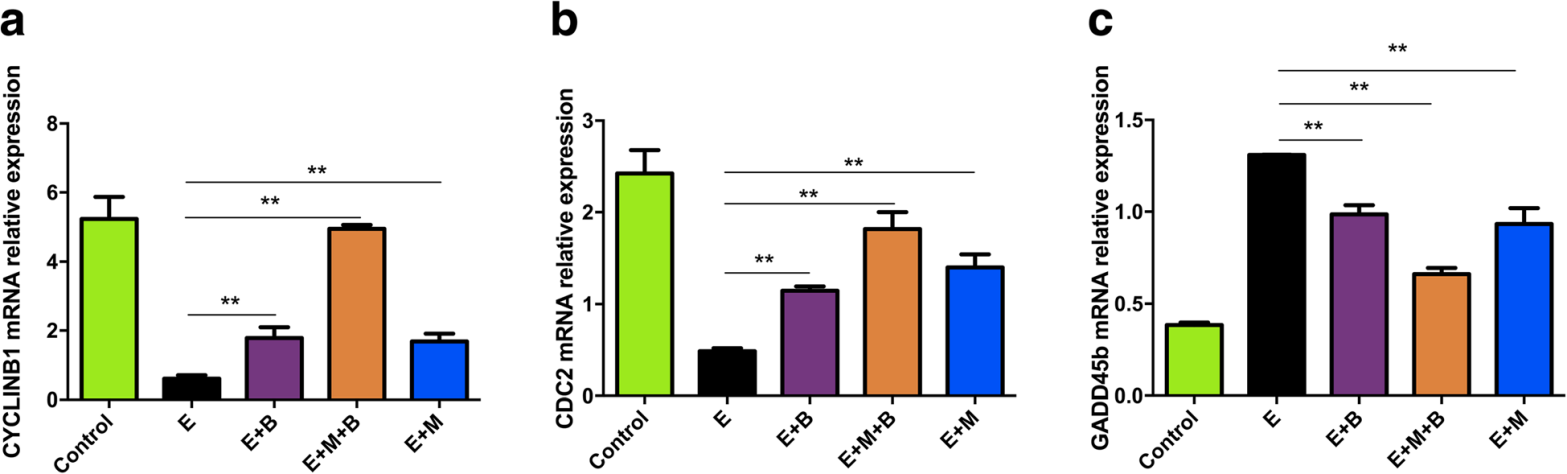
The effect of chemotherapy and different treatments on mRNA expressions. GAPDH was used as an endogenous control. **a** The quantitative graph of *Cyclinb1* expression. **b** The quantitative graph of *Cdc2* expression. **c** The quantitative graph of *Gadd45b* expression. *P < 0.05, **P < 0.01 v.s. E group

**Fig. 8:**
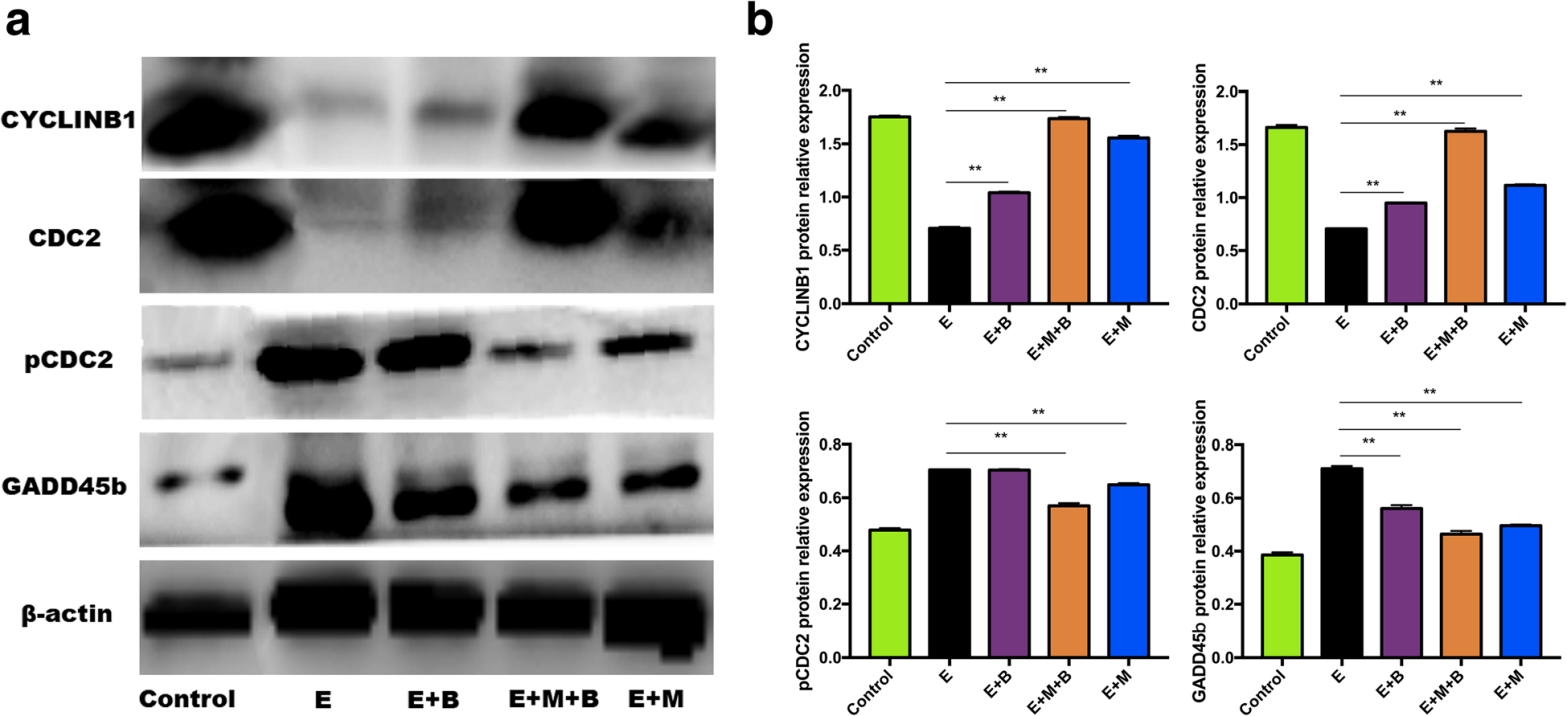
The effect of chemotherapy and different treatments on protein expressions. **a** GADD45b, CyclinB1, CDC2 and pCDC2 protein expressions were analyzed by Western blot analysis. β-actin was used as an endogenous control. **b** The quantitative graph of GADD45b, CyclinB1, CDC2 and pCDC2 protein expressions. *P < 0.05, **P < 0.01 v.s. E group

To detect whether the effect of MB-MSCs on GADD45b, CyclinB1 and CDC2 expressions in POF mice is consistent with the effect on damaged human GCs, their expressions were determined by RT-qPCR and western blot analysis. The results showed that GADD45b and pCDC2 mRNA and protein expressions were significantly higher in E group compared with those in control group (*P* < 0.01, Figs. 7 and 8). GADD45b expressions were decreased in E + M + B, E + M and E + B groups compared with that in E Group (*P* < 0.01, Figs. 7 and 8), and a more significant decrease in protein expression was observed in E + M + B group compared with that in E + M group and E + B group (*P* < 0.05, Fig. 8). pCDC2 protein expressions were decreased in E + M + B and E + M groups compared with that in E Group (*P* < 0.01, Fig. 8). E + M + B group showed a more significant decrease than E + M and E + B groups (*P* < 0.01, Fig. 8). No significant difference in pCDC2 protein expression was observed between E + B and E groups (*P* > 0.05, Fig. 8). CyclinB1 and CDC2 expressions were down-regulated in E group compared with the control group (*P* < 0.01, Fig. 8). CyclinB1 and CDC2 expressions in E + M + B, E + M and E + B groups were significantly increased compared with E group (*P* < 0.01, Fig. 8). E + M + B group showed a more significant increase than E + M and E + B groups (*P* < 0.01, Fig. 8).

## Discussion

Epirubicin is a first-line anti-tumor durg widely used in acute leukemia, lymphoma and breast cancer [3, 30, 31]. After treating human ovarian GCs with epirubicin in vitro, we have previously found that epirubicin inhibited the secretion of E2, progesterone, AMH, inhibin A and inhibin B and the proliferation of GCs, and promoted apoptosis of GCs. Studies have reported that cyclophosphamide may induce POF through PI3K/Akt/mTOR, Rictor/mTORC2/Akt/Foxo3a signalling axis [32, 33]. Cisplatin acts through PTEN/AKT/FOXO3a pathway [34]. Tripterygium glycosides act through serine/threonine kinase 11-p53-p21 signaling pathway [35]. In our previous study, a genome-wide transcriptional analysis using the Affymetrix GeneChip® identified 3599 significantly differentially expressed genes between granulosa cells treated/not treated with epirubicin groups, including 1962 upregulated genes and 1607 downregulated genes. The pathway analysis using the KEGG database showed that the cell cycle pathway was involved in 18 differentially expressed genes between granulosa cells treated/not treated with epirubicin groups. The microarray analysis results showed that of the three subtypes (GADD45a, GADD45b, and GADD45g) of GADD45, GADD45b was identified as a significantly differentially expressed gene. And we believe that epirubicin acts through activating GADD45b of the cell cycle pathway, which serves as one of the most important mechanisms of epirubicin-induced POF. In vitro experiments have also confirmed that epirubicin might inhibit the activity of CDC2/CyclinB1 complex by increasing the protein expression of GADD45b in the cell cycle pathway of ovarian GCs, resulting in inhibition of the proliferation of GCs and blocking in G2/M phase [22]. In this study, we found that epirubicin treatment resulted in a decrease in the number of primordial, primary, secondary and antral follicles; an increase in the number of atretic follicles; an increase in ovarian in situ apoptosis; a decrease in serum E2 and AMH levels; an increase in serum FSH levels; and the stagnation in diestrum or proestrum. This is probably because epirubicin can promote the expressions of GADD45b and pCDC2 but inhibit the expressions of CyclinB1 and CDC2.

Hormone replacement therapy (HRT) is one of the therapeutic options for POF. However, although HRT is capable of alleviating clinical symptoms of POF, but it is incapable of restoring the reproductive ability. Long-term oral administration of estrogen and/or progesterone can also increase the risk of venous thromboembolism (VTE), stroke, breast cancer, ovary cancer and other estrogen sensitive tumors [36–39]. Gonadotropin-releasing hormone angonist (GnRHa) is also thought to be effective in preventing chemotherapy induced POF, which is attributed to the reduction in ovarian perfusion and inhibition of follicles into the growth stage [40]. However, there are also reports that GnRHa is not associated with the decrease in POF risk [41, 42]. Assisted reproductive technology (ART) such as cryopreservation of embryos, oocytes and ovarian tissues is not mature enough to be widely used in clinic [43]. The pluripotent stem cells hold a promise in the treatment of POF due to their exceptional self-replication ability. MB-MSCs have the advantages of self-renewal, high proliferative potential, pluripotentiality and low immunogenicity [44, 45]. In early in vitro experiments, we found that MB-MSCs repaired epirubicin induced damage to human ovarian GCs by inhibiting GADD45b expression in the cell cycle pathway [22]. In epirubicin induced POF mice model, we found that MB-MSCs resulted in an increase in E2 and AMH levels and the number of preantral and antral follicles and a decrease in the number of atretic follicles, but the estrous cycle still showed no significant recovery on 28 d after MB-MSCs transplantation. Thus, we are expecting another way to improve damaged ovarian function with MB-MSCs. In Chinese traditional medicine, Shen governs reproduction, and thus invigorating Shen is helpful to improve the function of the reproductive system. BSTCR is a well-known traditional Chinese prescription capable of the expression of FSHR and IGF-1 mRNA, and thus it can promote the efficacy of gonadotropin to GCs and increase the reactivity of GCs to gonadotropin. We have previously found that BSTCR might recover the ovary reserve capacity via the BDNF pathway. BDNF is expressed in GCs and oocytes, and thus it plays a critical role in the development and maturation of oocytes. It is also involved in mitochondria assembly and motility, meiotic spindle configuration and cortical granule distribution during oocyte mutuation, prohibiting JNK and P38 activation under stress conditions, and increasing cell viability [23, 24]. We expect that BST CR can have an effect on the migration and survival of MB-MSCs act synergetically with MB-MSCs to improve damaged ovarian function.

We found that combined with BSTCR, MB-MSCs could result in the recovery of the estrous cycle, an increase in the number of promordial, primary, secondary and antral follicles, a decrease in the number of atretic follicles and ovarian cell apoptosis, an increase in serum E2 and AMH levels, and a reduction in serum FSH levels in epirubicin induced POF mice. However, although mice treated with MB-MSCs alone showed an increase in the number of follicles and sex hormone levels, the estrous cycle showed no obvious recovery. Thus, BSTCR was thought to act synergistically with MB-MSCs to improve damaged ovarian function.

As for the mechanism of MB-MSCs and BSTCR in improving ovarian function, according to the differentially expressed gene analysis previously, GADD45b might play a critical role in repairing epirubicin induced cell cycle arrest of GCs and promoting cell proliferation, and it could also have an effect on the CDC2/CyclinB1 complex and thus affect the cell cycle and proliferation of human ovarian GCs [22]. The RT-qPCR and western blot analysis showed that ovarian GADD45b and pCDC2 expressions were down-regulated, and CyclinB1 and CDC2 expressions were up-regulated in mice treated with MB-MSCs, BSTCR or MB-MSC combined with BSTCR. Importantly, MB-MSCs combined with BSTCR showed better therapeutic effects than BSTCR or MB-MSCs alone.

The GADD45 gene family encodes three related GADD45 proteins, including GADD45α, β and γ, with similar functions and sequences. Each gene is expressed in a number of mammal tissues such as heart, brain, spleen, lung, liver, skeletal muscle, kidney and testis [46]. GADD45 proteins are involved in many cell functions, including DNA repair, cell cycle control, cell survival or apoptosis, cell senescence, maintenance of genomic stability and genotoxic stress [47–50]. The members of GADD45 gene family have similar but not identical functions in different apoptosis and growth inhibitory pathways. GADD45α, β and γ have been suggested to mediate different signal pathways via different stimuli and finally mediate cell apoptosis and senescence. Their expression levels are generally low under normal conditions but significantly increased under stress [48]. In this study, we found that epirubicin treatment induced high expression of GADD45b. The function of GADD45 as a stress sensor is mediated via a complex interplay of physical interactions with other cellular proteins implicated in cell cycle regulation and the response of cells to stress, notably PCNA, p21, cdc2/cyclinB1, and the p38 and JNK stress response kinases [51]. The GADD45 isoforms have also been implicated in the G2/M cell cycle checkpoint in human and mouse cells [52]. In this study, the RT-qPCR and western blot analysis revealed an increase in GADD45b and pCDC2 expressions and a decrease in CyclinB1 and CDC2 expressions in mice treated with epirubicin, indicating that epirubicin may regulate cell cycle arrest of ovary cells by up-regulating GADD45b protein expression and down-regulating CDC2 and CyclinB1 expressions. However, the combined therapy resulted in a decrease in GADD45b and pCDC2 expressions and an increase in CyclinB1 and CDC2 expressions, indicating that MB-MSCs combined with BSTCR may repair epirubicin induced ovarian function damage by down-regulating GADD45b expressions and up-regulating CDC2 and CyclinB1 expressions. These results were in good agreement with our previous in vitro studies.

## Conclusions

Although the treatment of POF with BSTCR or MB-MSCs alone results in an increase in the number of follicles and sex hormone levels, the estrous cycle shows no obvious recovery. MB-MSCs combined with BSTCR could result in the recovery of the estrous cycle, an increase in the number of promordial, primary, secondary and antral follicles, a decrease in the number of atretic follicles and ovarian cell apoptosis, an increase in serum E2 and AMH levels, and a reduction in serum FSH levels in epirubicin induced POF mice. Importantly, it shows better therapeutic effects than BSTCR or MB-MSCs alone. This can be related to the inhibition of GADD45b expression and the promotion of CDC2 and CyclinB1 expressions. This study may be helpful for female patients of childbearing age with chemotherapy induced POF.

## Data Availability

The datasets used and/or analyzed during the current study are available from the corresponding authors on reasonable request.

## Abbreviations

AMH: Anti-Müllerian Hormone
ART: Assisted reproductive techniques
BCA: Bicinchoninic acid assay
BDNF: Brain derived neurotrophic factor
BSTCR: Bushen Tiaochong recipe
DAPI: 4′, 6-diamidino-2-phenylindole
DOR: Diminished ovarian reserve
E2: Estradiol
ELISA: Enzyme-linked immunosorbent
FSH: Follicle-Stimulating Hormone
GADD45: The growth arrest and DNA damage inducible 45
GFP: Green fluorescent protein
GnRHa: Gonadotropin-releasing hormone agonist
H&E staining: Hematoxylin and eosin staining
HRT: Hormone replacement therapy
IGF-1: Insulin-like growth factors − 1
MB-MSCs: Menstrual blood derived mesenchymal stem cells
MSCs: Mesenchymal stem cells
PBS: Phosphate-buffered saline
POF: Premature ovarian failure
PVDF: Polyvinylidene difluoride
SDS: Sodium dodecyl sulfate
TUNEL: Terminal deoxynucleotidyltransferase-mediated deoxy-UTP nick end labeling
VTE: Venous thromboembolism
